# Genome Sequence of *Pseudomonas chlororaphis* Strain 189

**DOI:** 10.1128/genomeA.00581-16

**Published:** 2016-06-23

**Authors:** Jennifer Town, Patrice Audy, Susan M. Boyetchko, Tim J. Dumonceaux

**Affiliations:** a Saskatoon Research and Development Centre, Agriculture and Agri-Food Canada, Saskatoon, Saskatchewan, Canada; b Department of Veterinary Microbiology, University of Saskatchewan, Saskatoon, Saskatchewan, Canada; c Québec Research and Development Centre, Agriculture and Agri-Food Canada, Québec, Québec, Canada

## Abstract

*Pseudomonas chlororaphis* strain 189 is a potent inhibitor of the growth of the potato pathogen *Phytophthora infestans*. We determined the complete, finished sequence of the 6.8-Mbp genome of this strain, consisting of a single contiguous molecule. Strain 189 is closely related to previously sequenced strains of *P. chlororaphis*.

## GENOME ANNOUNCEMENT

*Pseudomonas chlororaphis* strain 189 was isolated from soil near Golden Prairie, Saskatchewan, Canada. This organism has been studied as a possible biocontrol agent for the potato pathogen *Phytophthora infestans*. To elucidate the potential metabolic pathways this organism may use to exhibit its growth-inhibiting phenotype, we determined its complete genomic sequence.

*P. chlororaphis* 189 was grown at 22°C in a rotary shaker for 24 h in yeast extract glucose medium (2.0 g/L yeast extract, 2.5 g/L glucose, 0.4 mM MgSO_4_·7H_2_O, 0.09 mM MnSO_4_·H_2_O, 0.85 mM NaCl, 0.017 mM FeSO_4_·7H_2_O, 1.84 mM KH_2_PO_4_, and 1.43 mM K_2_HPO_4_). Genomic DNA was purified from 1 mL of overnight culture using the Wizard gDNA extraction kit (Promega, Madison, WI, USA) and sequenced on the MiSeq platform using the mate-pair protocol (Illumina), generating 2.8 Mb of mate-pair reads. An additional 8-kb insert paired-end sequencing run was performed based on the paired-end rapid library preparation protocol for Titanium chemistry (Roche, March 2012), with modifications as described (1), generating 166,514 paired-end reads with an estimated pair distance of 5,641 ± 1,410 bp. Illumina reads were assembled using SOAPdenovo2 version 2.01 with *k*-mer size 127 and map length 34. The resulting 957 contigs (*N*_50_ 33,267 bp) were split into 500-bp pieces with a 200-bp overlap using EMBOSS splitter, combined with the Roche paired-end reads, and reassembled using Newbler version 3.0. Gaps in the sequence were filled using the GapCloser tool for SOAPdenovo2, along with PCR and Sanger sequencing. Assembly of all sequencing data together produced a finished (2) 6.8-Mbp genome sequence with 152× coverage, featuring a single scaffold with no gaps and no evidence of any plasmids. Sequence data were annotated using the Prokaryotic Genome Annotation Pipeline version 3.1 (NCBI).

The genome of *P. chlororaphis* 189 contained 6,837,781 bp (62.74% G/C); 6,025 genes and 5,934 protein-encoding genes were observed, along with 6 genes encoding 5S rRNA, 5 genes encoding 16S rRNA, 5 genes encoding 23S rRNA, and 71 tRNA-encoding genes. Moreover, 2,075 clusters of orthologous groups were identified by annotation using the Integrated Microbial Genomes portal (https://img.jgi.doe.gov/cgi-bin/mer/main.cgi).

Examination of the *cpn60* sequence (3) of *P. chlororaphis* 189 suggested that this strain was most closely related to *P. chlororaphis* subsp. *aureofaciens* 30-84 (NZ_CM001559.1), with a nucleotide identity of 99.6%. Consistent with this observation, determination of the genome-level average nucleotide identity (4) with 14 other genomes from this species revealed that *P. chlororaphis* 189 shared the highest genome sequence identity (98.36%) with this strain. Several other strains of *P. chlororaphis* exhibiting biocontrol phenotypes, including PA23 (5), had genome similarity metrics that placed them in the same species as *P. chlororaphis* 189. Like PA23, P*. chlororaphis* 189 contained an array of biosynthetic pathways capable of producing metabolites involved in biocontrol, including hydrogen cyanide (6), phenazine (7), pyocin (8), pyrroloquinoline quinone (9), and cell wall degradative enzymes. Genes conferring the ability to produce surfactants and form biofilms (10) were also found.

### Nucleotide sequence accession numbers.

The sequence data for this complete genome has been deposited at DDBJ/EMBL/GenBank under accession number CP014867.
